# Trajectories of body mass index and risk for coronary heart disease: A 38-year follow-up study

**DOI:** 10.1371/journal.pone.0258395

**Published:** 2021-10-07

**Authors:** Susanna Calling, Sven-Erik Johansson, Veronica Milos Nymberg, Jan Sundquist, Kristina Sundquist

**Affiliations:** Center for Primary Health Care Research, Department of Clinical Sciences in Malmö, Lund University, Region Skåne, Sweden; McMaster University, CANADA

## Abstract

**Objective:**

Obesity is a well-known risk factor for coronary heart disease (CHD), but there is little evidence on the effect of long-term trajectories of body mass index (BMI) over the life course. By using repeated assessments, the aim was to study the risk of CHD in adults during 38 years in different trajectories of BMI.

**Methods:**

A sample of 2129 men and women, aged 20–59 years at baseline, took part in four repeated interviews between 1980 and 2005. Data on BMI, medical history, lifestyle and socioeconomy were collected. Based on the World Health Organization categories of BMI, life course trajectories of stable normal weight, stable overweight, stable obesity, increasing BMI and fluctuating BMI were created. The individuals were followed through national registers for first hospitalization of CHD (389 events) until the end of 2017, and Hazard Ratios (HRs) were calculated, adjusted for age, sex, socioeconomic factors, lifestyle factors and metabolic comorbidities.

**Results:**

Stable normal weight in all assessments was the reference group. Those who had an increase in BMI from normal weight in the first assessment to overweight or obesity in later assessments had no increased risk of CHD, HR 1.04 (95% CI: 0.70–1.53). The HR for individuals with fluctuating BMI was 1.25 (0.97–1.61), for stable overweight 1.43 (1.03–1.98), for stable obesity 1.50 (0.92–2.55), and for stable overweight *or* obesity 1.45 (1.07–1.97), after full adjustments.

**Conclusion:**

Having a stable overweight or obesity throughout adult life was associated with increased CHD risk but changing from normal weight at baseline to overweight or obesity was not associated with increased CHD risk. Prevention of obesity early in life may be particularly important to reduce CHD risk.

## Introduction

The prevalence of obesity has increased globally in both adults and children and constitutes a major public health problem because of the associated chronic diseases, premature mortality and significant societal costs [1, 2]. In Sweden, overweight and obesity have increased during recent decades, although a plateau trend has been indicated [3, 4]. Between 1980 and 2005, mean BMI increased from 24.1 to 25.5 kg/m^2^ in men and from 23.1 to 24.3 kg/m^2^ in women [3].

Among other health-related consequences, overweight and obesity are well-established risk factors for coronary heart disease (CHD) in both men and women [5–7]. This is partly explained by metabolic disorders such as hypertension, type 2 diabetes mellitus and dyslipidemia, but obesity has also been shown to be a risk factor independently of metabolic risk factors [2]. CHD is still the leading cause of death in both men and women globally. Despite a generally decreasing trend due to better treatment and secondary prevention, there are a few reports about increased rates of CHD in, e.g., women in the USA [8, 9].

In addition to the association between obesity and CHD, previous research has shown that weight gain during adulthood is associated with an increased risk of both cardiovascular risk factors and incidence of CHD [10–12]. However, a study from the UK found that weight gain after 18 years of age was not associated with CHD per se, but with smoking habits, and the highest weight-gain was found in ex-smokers [12]. Moreover, several studies have shown that childhood obesity is associated with a deteriorated cardiometabolic profile in adolescence and young adulthood [13, 14]. Elevated BMI in both adolescence and adulthood seems to be associated with angiography-proven CHD [15]. However, few studies have analyzed the cardiovascular effects of long-term trajectories of BMI throughout life. A recent Chinese study of children and adolescents aged 6–18 years showed that a trajectory of high-increasing BMI was associated with a 3-fold increased risk of hypertension, type 2 diabetes mellitus and dyslipidemia, during 30 years of follow-up [16]. Similar results were shown in a study of Finnish individuals [17]. These two studies were limited to cardiovascular risk factors and not CHD events, and the follow-up ended when the participants were young/middle aged, before the point when the majority of CHD cases usually occur. Another recent study examined different patterns of BMI trajectories in Chinese individuals aged 6–60 years during 20 years of follow-up [18]. The study showed that individuals with stable normal weight throughout this period had the lowest risk of developing cardiovascular risk factors (high blood pressure, high blood glucose, dyslipidemia) and that individuals who had increasing BMI from overweight to obesity had the highest risk.

Consequently, earlier studies about BMI trajectories over the life course and effects on cardiovascular risks have been limited by shorter follow-up [17], no CHD events as endpoints [16–18], or recall bias [11, 12, 19]. Few studies have examined the risk of CHD in relation to long term trajectories of BMI through the life course, and cohorts with repeated values of weight and height over several decades are scarce. Clinical BMI guidelines are mostly based on the classification made by the World Health Organization (WHO) [20], i.e., normal weight, overweight and obesity, which are therefore used in the present study.

The novelty with the present study is the access to four repeated assessments of BMI in a Swedish population cohort over 26 years, with an additional follow-up of CHD events of 12 years, from young adulthood to older age. The aim was to study the risk of CHD in this cohort by different BMI trajectories based on the WHO categories, i.e., stable normal weight, stable overweight, stable obesity, increasing BMI and fluctuating BMI, over the total follow-up of 38 years.

## Materials and methods

### The Swedish Annual Level of Living Survey

The Swedish Annual Level of Living Survey (SALLS) has been conducted annually since 1974 by Statistics Sweden and has been described in detail previously [3, 21]. In short, the survey includes a nationally representative random sample of Sweden’s population aged 16–84 years, drawn from the Swedish Total Population Register. Between 1980 and 2005, assessments were made every eighth year with a repeated set of survey questions to the same individuals, to make it possible to follow changes over time. The individuals were invited by letter and face-to-face interviews were conducted in the respondents’ homes by professional interviewers from Statistics Sweden. In the present study, we included 3282 women and men who had taken part in three or four repeated assessments (1980/81, 1988/89, 1996/97 and 2004/05) and were aged between 20 and 59 years at the first assessment.

### Follow-up and outcome variable

The individuals were followed from the day of the first assessment for first hospitalization of CHD, identified from the National Patient Register, or until death, emigration or the end of the study on Dec 31^st^, 2017 (total follow-up 38 years). The diagnosis of CHD was defined according to the International Classification of Diseases (ICD), i.e. I20—I25 (ICD-10) or 410–414 (ICD-9). Individuals with CHD before baseline (n = 6) were excluded. No values were included after a CHD diagnosis.

### BMI

Weight and height were self-reported, and BMI was calculated as weight (kg)/height (m)^2^. BMI was used in three ways: 1) continuous BMI, 2) BMI categories based on the classification of WHO [20]; normal weight (NW, BMI <25 kg/m^2^), overweight (OW, BMI 25.0–29.9 kg/m^2^) and obesity (OB, BMI ≥30 kg/m^2^), and 3) BMI trajectories, which were defined as follows:

- stable normal weight (NW in all assessments)
- stable overweight (OW in all assessments)
- stable obesity (OB in all assessments)
- increasing BMI (NW in the first assessment and OW or OB in later assessments)
- fluctuating BMI (all others, i.e. fluctuating BMI over the assessments).

The categories and trajectories were generated according to the BMI values at the date of assessment and adjusted for age as a time-varying variable.

### Covariates

The variables listed below were included as time-varying covariates (apart from sex) [22], and selected based on previous literature of the association between BMI and CHD [8, 23, 24]. All variables were measured at each assessment, and the majority of the variables were based on the interviews, but diabetes mellitus type 2, hypertension and dyslipidemia were based on ICD diagnoses from the National Patient Register (hospital care 1980–2017 and outpatient care 2001–2017) and almost nationwide primary care data from the Swedish regions (1997–2017).

*Age* was assessed at the time of interview and was categorized in 8-year intervals, based on the assessment periods, see Table 1.

**Table 1:** The distribution (%) and means (age and BMI) of the included variable by period of time.

Variable	Level	Year			
Assessment period		1980/81	1988/89	1996/97	2004/05
n		2129	2129	2129	2129
Sex (%)	Men	47.2	47.2	47.2	47.2
	Women	52.8	52.8	52.8	52.8
Age (years)	Mean	36.9	44.9	52.9	60.9
	Range	20–59	28–67	36–75	44–83
Age (%)	20–27	23.4	-	-	-
	28–35	25.2	23.4	-	-
	36–43	22.7	25.2	23.4	-
	44–51	16.9	22.7	25.2	23.4
	52–59	12.2	16.9	22.7	25.2
	60–67	-	12.2	16.9	22.7
	68–75	-	-	12.2	16.9
	76–83	-	-	-	12.2
BMI	Mean	23.3	24.3	25.2	25.8
	95% CI	23.1–23.4	24.0–24.3	25.1–25.4	25.6–25.9
BMI	≥14 - <25 kg/m^2^	75.2	64.8	50.9	45.0
category (%)	≥25 - <30 kg/m^2^	21.5	29.0	40.6	42.2
	≥30 - <60 kg/m^2^	3.3	6.2	8.5	12.8
BMI (mean)	NW+NW+NW+NW^a^	20.9	21.4	22.0	22.2
trajectory	OW+OW+OW+OW^b^	26.6	27.2	27.6	27.8
	OB+OB+OB+OB^c^	32.8	34.0	35.2	35.2
	NW 80/81+ (OW or OB)^d^	23.7	26.4	27.8	28.6
	Fluctuating^e^	24.0	24.9	26.4	27.3
Education (%)	Low	32.7	28.2	27.4	28.6
	Medium	44.7	44.5	45.1	43.0
	High	22.6	27.3	27.5	28.4
Urbanization (%)	Three largest cities	28.6	28.8	28.1	27.4
	Medium-sized towns	32.6	34.6	37.3	37.6
	Small towns/rural area	38.8	36.6	34.6	35.0
Marital	Single-living	23.8	19.1	22.1	27.8
status (%)	Cohabiting/married	76.2	80.9	77.9	72.2
Smoking (%)	Non-smokers	42.1	42.3	42.6	37.7
	Ex-smokers	25.1	30.8	36.7	46.6
	Daily smokers	32.8	26.9	20.7	15.7
Exercise (%)	Almost none	49.0	50.0	46.4	43.3
	≥ Once a week	51.0	50.0	53.6	56.7
		**Totals**			
Diabetes* (%)	No	88.3			
	Yes	11.7			
Hypertension* (%)	No	58.6			
	Yes	41.4			
Dyslipidemia* (%)	No	80.9			
	Yes	13.4			

^a^ Normal weight in all assessments

^b^ overweight in all assessments

^c^ obesity in all assessments

^d^normal weight in the first assessment 1980/81 and overweight or obesity in later assessments

^e^ fluctuating BMI, all others.

*Totals mean percentage for the entire follow-up period and occurring before CHD.

*Sex* was analyzed as women and men.

*Educational level* was self-reported and categorized as 1) high (≥12 years of school), 2) medium (10–11 years) and 3) low (≤9 years).

*Urbanization* was defined as residence in 1) the three largest cities in Sweden, 2) medium-sized towns (population >90.000 individuals) and small towns/rural areas (population <90.000).

*Marital status* was dichotomized as single or cohabiting/married.

*Smoking* was dichotomized as 1) non-smokers, which comprised never smokers and former smokers, and 2) daily smokers.

*Exercise* was based on a question about physical activity during leisure time, with five options from “basically nothing” to “regular rather strenuous activity at least twice a week, and dichotomized into 1) almost none, options 1–2, and 2) at least once a week, options 3–5.

*Diabetes mellitus*: yes/no (ICD-10 E11, ICD-9 250)

*Hypertension*: yes/no (ICD-10 I10-I15, ICD-9 401–405)

*Dyslipidemia*: yes/no (ICD-10 E78, ICD-9 272)

### Ethical approval

The study was approved by The Regional Ethical Review Board in Stockholm (No. 2012/95 approved Feb 6, 2013, and 2015/830 approved Dec 3, 2015).

### Statistics

In this study, we included only those individuals who participated in all four interviews/assessments. Missing values for any of the analyzed variables in any of these assessments (1–3%) were replaced by the closest available assessment by the method of carrying forward or backward, according to previous literature [25]. All variables except for sex were included as time-varying variables. Descriptive statistics were used to show the distribution and means (BMI and age, Table 1) of the included variables for each survey year, as well as number of CHD events (Table 2).

**Table 2:** Number of CHD and sex- and age-adjusted hazard ratios (HR) with 95% confidence intervals (CI) for all included covariates, n = 2129 (4 assessments).

Variable	Level	CHD, n	Sex- and age-adjusted models, HR (CI)
Sex	Men	235	2.04 (1.67–2.50)
	Women	154	1 (ref)
Age	Categorized		Not shown*
BMI	Continuous	389	1.06 (1.03–1.08)
BMI	≥14 - <25 kg/m^2^	146	1 (ref)
category	≥25 - <30 kg/m^2^	182	1.32 (1.06–1.64)
	≥30 - <60 kg/m^2^	61	1.83 (1.35–2.48)
BMI	NW+NW+NW+NW^a^	102	1 (ref)
trajectory	OW+OW+OW+OW^b^	59	1.62 (1.17–2.23)
	OB+OB+OB+OB^c^	20	2.42 (1.53–3.84)
	NW 80/81+(OW or OB)^d^	39	1.18 (0.81–1.71)
	Fluctuating BMI^e^	169	1.40 (1.10–1.79)
Education	Low	161	1 (ref)
	Medium	154	0.87 (0.69–1.09)
	High	74	0.74 (0.49–1.02)
Urbanization	Three largest cities	89	1 (ref)
	Medium-sized towns	134	1.18 (0.90–1.54)
	Small towns/rural area	166	1.49 (1.15–1.93)
Marital	Single-living	106	1 (ref)
status	Cohabiting/married	283	0.88 (0.69–1.11)
Smoking	Non-smokers	142	1 (ref)
	Former smokers	173	1.29 (1.03–1.62)
	Daily smokers	74	1.63 (1.23–2.17)
Exercise	Almost none	185	1 (ref)
	≥ Once a week	204	0.93 (0.76–1.14)
Diabetes	No	333	1 (ref)
	Yes	56	2.48 (1.83–3.36)
Hypertension	No	241	1 (ref)
	Yes	148	2.45 (1.91–3.13)
Dyslipidemia	No	341	1 (ref)
	Yes	48	2.16 (1.54–3.03)

^a^ Normal weight in all assessments

^b^ overweight in all assessments

^c^ obesity in all assessments

^d^normal weight in the first assessment 1980/81 and overweight or obesity in later assessments

^e^ fluctuating BMI, all others.

*HR increased by increasing age categories.

All models satisfied the proportional hazards assumption (p>0.10).

We applied Cox regression models to calculate hazard ratios (HRs) with 95% confidence intervals (CIs) for the associations between BMI and CHD, adjusted for sex and age, and in a full model adjusted for all variables. In the full model, the non-significant covariates were included as strata and therefore no HRs were obtained for these variables in the full models. BMI was analyzed as a continuous variable, in the three categories, and as BMI trajectories. We considered differences as significant when *p* was <0.05.

We used the estat phtest command in STATA for individual covariates and globally, using scaled Schoenfeld residuals. The null hypothesis of zero slope is equivalent to testing that the log hazard-ratio function is constant over time. Thus, rejection of the null hypothesis of a zero slope indicates deviation from the proportional hazards. All models satisfied the proportional hazards assumption (p>0.10).

We tested for linearity by including BMI squared in the continuous model and it was not significant; thus, we judge that the relationship between BMI and CHD is linear.

No meaningful interactions were found between BMI and any of the covariates. We also tested the interactions between sex and all the other covariates, and none was found.

STATA version 16.1 was used for the statistical analyses [26].

## Results

The study population was between 20 and 59 years old (mean age 36.9 years) at the first assessment in 1980/81, and 44–83 years old (mean age 60.9 years) at the last assessment in 2004/05 (Table 1). Mean BMI ranged from 23.3 kg/m^2^ to 25.8 kg/m^2^ between the first and last assessment, and the proportion of individuals with obesity (≥ 30 kg/m^2^) increased from 3.3% to 12.8%. The number of individuals in each trajectory were as follows: stable normal weight: 815, stable overweight: 179, stable obesity: 47, increasing BMI: 234 and fluctuating BMI: 854. For all the defined BMI trajectories, mean BMI increased over time. In the total study population, the proportion of individuals who had a diagnosis of a metabolic comorbidity at any of the assessments was 11.7% for diabetes, 41.4% for hypertension and 13.4% for dyslipidemia. Please note that the proportions do not correspond to prevalence rates at a specific time point as the study period was 38 years.

**Fig 1:**
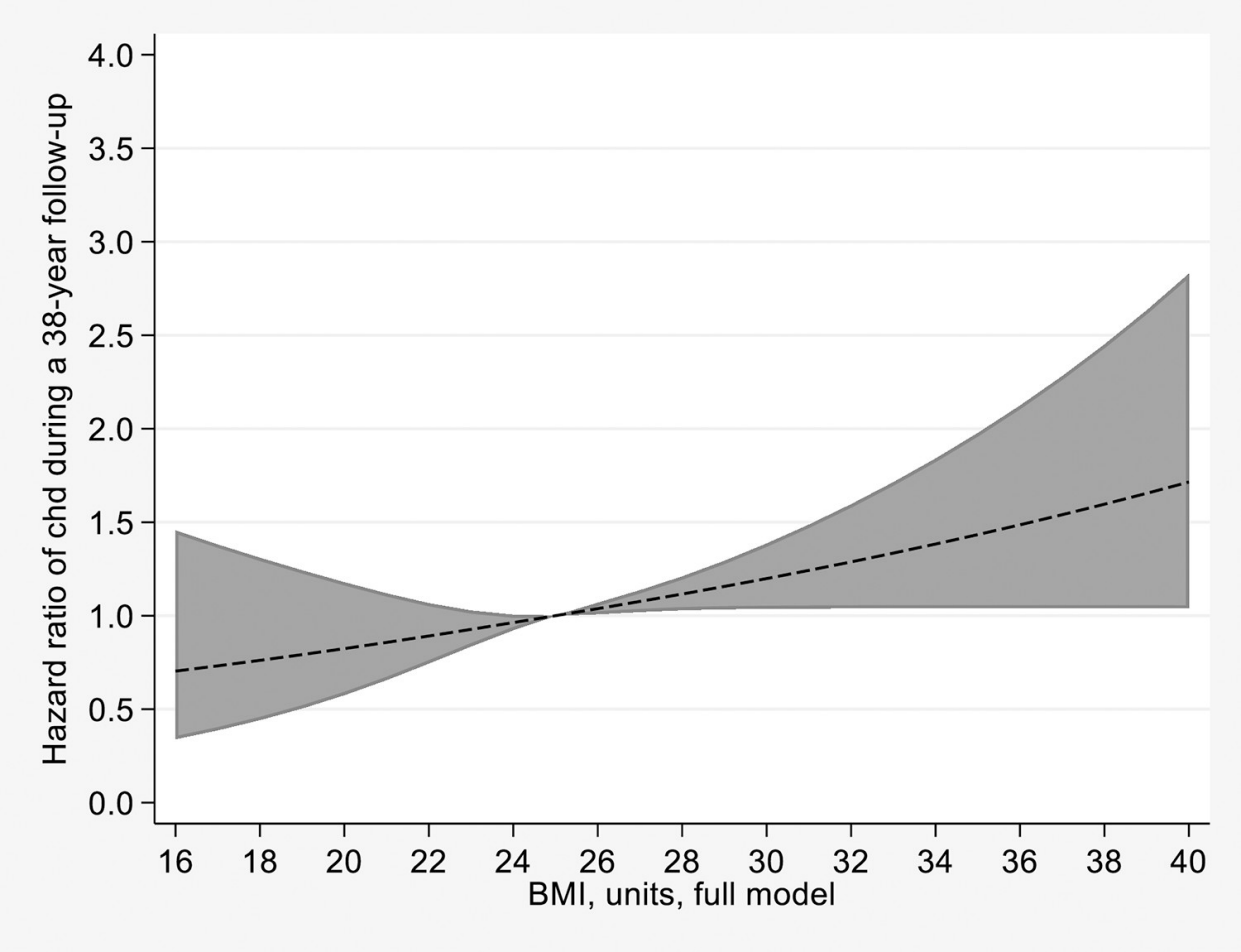
HR of CHD during 38-year follow-up, full model (Model 1), body mass index (BMI) continuous. HR, Hazard ratio; CHD, coronary heart disease, BMI, body mass index. Reference level (HR = 1) is BMI = 25.

During the follow-up, a total of 235 men and 154 women had a CHD event (Table 2). Analyzing BMI continuously showed an increased risk for CHD when BMI increased with an HR of 1.06 (95% CI: 1.03–1.08) as shown in Table 2 (sex- and age-adjusted model) and an almost perfect linearity as shown in Fig 1 (full model). Having an overweight or obesity was associated with HRs of 1.32 (1.06–1.64) and 1.83 (1.35–2.48), respectively, in sex- and age-adjusted models. Hypertension, diabetes, dyslipidemia, smoking and urbanization were also associated with CHD in the age- and sex adjusted model.

Table 3 shows the number of CHD and the distribution of CHD in each BMI trajectory, as well as the HRs for CHD for the three measures of BMI (continuous BMI, BMI categories and BMI trajectories), adjusted for all the potential covariates. The following variables were no longer significantly associated with CHD in any of the three models and are therefore included as strata and not shown in the table: educational level, marital status and exercise. Continuous BMI was still statistically significantly associated with an increased risk of CHD, as well as the BMI category obesity, whereas overweight was not. For BMI trajectories, having a stable normal weight in all assessments was used as the reference group. Having a stable overweight was associated with a HR of 1.43 (95% CI: 1.03–1.98) and the HR for stable obesity was 1.53 (0.92–2.55), after full adjustments. When merging stable overweight and stable obesity into one trajectory, the HR was 1.45 (1.07–1.97). Those who had an increasing BMI from normal weight in the first assessment to overweight or obesity had no increased risk of CHD, HR 1.04 (0.70–1.53). Finally, the HR for individuals with fluctuating BMI was 1.25 (0.97–1.61).

**Table 3:** Hazard ratios (HR) with 95% confidence interval (CI) for models 1–3, n = 2129. All models are adjusted for age (not shown).

Variable	Level	Model 1	Model 2	Model 3
Sex	Man	2.00 (1.62–2.48)	1.99 (1.61–2.47)	1.96 (1.58–2.42)
	Woman	1 (ref)	1 (ref)	1 (ref)
BMI	Continuous	1.04 (1.01–1.06)	-	-
BMI	≥14 - <25 kg/m^2^	-	1 (ref)	-
category	≥25 - <30 kg/m^2^	-	1.27 (1.01–1.59)	-
	≥30 - <60 kg/m^2^	-	1.40 (1.00–1.96)	-
BMI	NW+NW+NW+NW ^a^	-	-	1 (ref)
trajectory	OW+OW+OW+OW^b^	-	-	1.43 (1.03–1.98)
	OB+OB+OB+OB^c^	-	-	1.53 (0.92–2.55)
	NW 80/81+(OW or OB)^d^	-	-	1.04 (0.70–1.53)
	Fluctuating^e^	-	-	1.25 (0.97–1.61)
Urbanization	Three largest cities	1 (ref)	1 (ref)	1 (ref)
	Medium-sized towns	1.26 (0.96–1.65)	1.24 (0.95–1.63)	1.25 (0.95–1.64)
	Small towns/rural area	1.53 (1.18–1.99)	1.53 (1.18–1.99)	1.53 (1.18–1.99)
Smoking	Non-smokers	1 (ref)	1 (ref)	1 (ref)
	Former smokers	1.31 (1.04–1.65)	1.31 (1.04–1.65)	1.33 (1.06–1.67)
	Daily smokers	1.74 (1.30–2.32)	1.71 (1.28–2.29)	1.69 (1.26–2.26)
Diabetes	No	1 (ref)	1 (ref)	1 (ref)
	Yes	1.60 (1.13–2.28)	1.66 (1.16–2.36)	1.67 (1.18–2.36)
Hypertension	No	1 (ref)	1 (ref)	1 (ref)
	Yes	2.02 (1.53–2.67)	2.04 (1.54–2.69)	2.06 (1.56–2.72)
Dyslipidemia	No	1 (ref)	1 (ref)	1 (ref)
	Yes	1.42 (0.98–2.06)	1.42 (0.98–2.06)	1.42 (0.98–2.06)

Model 1, BMI continuous; Model 2, BMI categories; Model 3, BMI trajectories.

^a^ Normal weight in all assessments

^b^ overweight in all assessments

^c^ obesity in all assessments

^d^normal weight in the first assessment 1980/81 and later increasing BMI

^e^ fluctuating BMI, all others.

The non-significant variables Education, Marital status, and Exercise are included in all models as strata, whereby no HRs are obtained. All models satisfied the proportional hazards assumption.

## Discussion

The results of this long-term follow up of CHD risk in relation to BMI trajectories, created on the basis of four repeated BMI assessments in adults, showed that having a stable overweight or obesity throughout 26 years was associated with a significantly increased risk of CHD, adjusted for age and sex, while increasing BMI from normal weight to overweight/obesity was not. After further adjustments for socioeconomic factors, smoking, exercise and metabolic comorbidities, the statistical significance disappeared for stable obesity. However, the risk pattern was similar, and the insignificance is probably explained by the small number of individuals in this group as the association was significant in the combined trajectory overweight/obesity. Moreover, this indicates that the covariates had a significant impact on the association. The study also confirmed previous findings that BMI is a risk factor for CHD, both when analyzed as a continuous variable and as BMI categories [5–7], and that obesity in early adulthood is a strong predictor for CHD [12]. To the best of our knowledge, this is the first study that analyzed BMI trajectories during a 26-year period and included a 38-year follow-up throughout old age. Previous studies on BMI trajectories have mostly been limited by a shorter follow-up or recall bias [11, 12, 16–18]. Some of our results are in-line with previous research that has found that a high BMI in young adulthood is related to an increased risk of CHD later in life [12]. Earlier studies have also found that increasing BMI from childhood to adulthood is associated with a deteriorated cardiometabolic risk profile [16–18]. However, it has also been suggested that weight gain after 18 years of age bears little relation with subsequent CHD [12].

The findings indicate that the obesity-related pathogenetic process of atherosclerosis and subsequent development of CHD is complex and may vary during the life course. Long-term overweight and obesity in adults were associated with a subsequent increased risk of CHD in our study but a weight increase was not. Previous research has found that increased BMI in childhood is associated with increased risk of CHD in adulthood [27]. An Australian study found that increased BMI in childhood was associated with increased risk of metabolic cardiovascular risk factors (hypertension, type 2 diabetes, dyslipidemia and carotid intima media thickness) in adulthood [17]. These increased risks were reduced if the high BMI was reversed in adulthood; however, this risk reduction could not be observed for high-risk carotid intima media thickness, suggesting that obesity in childhood may irreversibly alter the arterial structure. Moreover, it has been shown that children with obesity already present with hypertension, dyslipidemia and impaired glucose tolerance and vascular abnormalities [28, 29]. This strengthens the importance of intervention and prevention towards childhood obesity. It is also possible that different types of obesity may manifest at different ages. For example, a study from China showed that different types of obesity were differentially associated with CVD risk factors [30]. Although we had no data on the different types of obesity in the present study, it is possible that obesity that occurs in earlier ages may be more harmful than obesity that occurs in relation to aging.

In our study, individuals who had a normal weight at the first assessment and later developed overweight or obesity had no increased risk of CHD. The findings are interesting and suggest that the most important alterations in metabolic profile and arterial function may take place in early life. It is known that the process of atherosclerosis starts already in early childhood [31]. It is possible that the decreased rates of current smoking in the later BMI assessments may have played a role in this group, as weight gain is more common in ex-smokers [12]. However, our results remained after adjustment for smoking status and no interaction was found between BMI and smoking. In adulthood, cardiometabolic multimorbidity is common in individuals with overweight and obesity. A large cohort study, with pooled data from USA and Europe, showed that the risk of cardiometabolic multimorbidity was doubled in individuals with overweight and increased more than ten times in people with severe obesity compared with individuals with a normal BMI [32]. The association between obesity and impaired endothelial function seems, however, to be of less importance in older individuals [33]. A Swedish study found that the association between high BMI and risk for CHD disappeared from age 70 and older [34]. Numerous studies have discussed an “obesity paradox” in which individuals with obesity with established cardiovascular disease and hypertension have a better prognosis than individuals without obesity with the same risk factors [35].

The group “fluctuating BMI” was most likely heterogeneous. In the present study, these individuals had a moderately increased risk of CHD which did not remain after full adjustment. These individuals’ BMI might have fluctuated more between the assessments compared to individuals in other groups, but we have no data on this. It is also possible that some individuals had fluctuating BMI but did not cross the cut-off for normal weight, overweight or obesity. A study of individuals with established CHD found that fluctuating body weight was associated with higher mortality and increased risk of CHD events [36]. Another study found that both weight gain and weight loss were associated with increased risk of atrial fibrillation [37].

The present results support the importance of prevention and intervention against overweight and obesity early in life. Several randomized control trials have shown that lifestyle interventions resulting in weight loss improve cardiovascular risk factors such as blood pressure and cholesterol levels [38]. Moreover, it has been shown that the cardiovascular risk may be reduced by physical activity even in individuals with obesity [39]. Individuals with obesity constitute a heterogeneous population and the concept of “metabolically healthy obesity” has been discussed frequently in existing medical literature [39–41]. However, a cohort study, which was performed among Chinese adults, showed that individuals with metabolically healthy obesity had an increased risk of major cardiovascular events and that 40% of them transitioned to metabolically unhealthy obesity health status during a 10-year follow up [40]. A recent large case-control cohort analysis found that, irrespective of metabolic health, people with overweight and obesity had higher CHD risk than their counterparts with normal weight, thus challenging the concept of “metabolically healthy obesity” [41]. The results are consistent with findings in another comprehensive cohort study concluding that even individuals with metabolically healthy obesity had a higher risk of CHD [42].

### Limitations and strengths

One limitation of the present study was that most of the variables were self-reported. Self-reported data may be biased by willingness to report and the social context of the individuals [43]. This may have led to underreporting of overweight and obesity, but the potential bias should not have changed between the assessments. Moreover, weight and height were the only anthropometric data, and additional data on waist and/or hip circumference would be preferable. Another limitation is that several variables as well as the cardiovascular risk may change between the assessments. BMI might have increased within a trajectory without changing from one WHO category to another. As previously mentioned, we did not analyze BMI changes within the categories of normal weight, overweight and obesity. However, as these categories are mostly used in clinical settings, we believe they are more suitable for the implications of the study in treatment and prevention of cardiovascular risk in patients. In long-term follow-up cohort studies, there is a possibility of reverse causation. In the present study, all registered CHD events occurred after the last assessment. This means that all participants included in the present study took part in all assessments. The present design does not allow for testing whether the associations differed during the first years of follow-up, as the participants were relatively young at the start of follow-up and very few CHD events occurred during the first years of follow-up.

The limitations are balanced by several strengths of the study. A key strength is the long study period with almost complete data of four repeated assessments and total follow-up of 38 years, that enabled us to study the BMI trajectories during a long time period and analyze the impact on later CHD. As the data were collected at each assessment, there was little risk for recall bias. Another strength is the large nationally representative random sample of individuals with comprehensive data, including several potential confounders. The study surveys were conducted as face-to-face interviews in the respondents’ homes and the high reliability of the survey questions has previously been tested in a subsample [3, 44].

## Conclusions

Having a stable overweight or obesity throughout adult life was associated with increased CHD risk but changing from normal weight to overweight or obesity did not seem to be associated with increased CHD risk. Prevention of obesity early in life may be important to reduce future cardiovascular risk.
